# Years of life lost in cutaneous squamous cell carcinoma: analysis of a prospective cohort of 1400 patients

**DOI:** 10.1007/s00432-026-06515-8

**Published:** 2026-05-25

**Authors:** Klaus Dietz, Thomas K. Eigentler, Helmut Breuninger

**Affiliations:** 1 Department of Medical Biometry (Emeritus), University of Tübingen, Tübingen, Germany; 2 Department of Dermatology, Venereology and Allergology, Charité—Universitätsmedizin Berlin, Luisenstrasse 65, 10117 Berlin, Germany; 3 Department of Dermatology, University Hospital Tübingen, Tübingen, Germany

**Keywords:** Cutaneous squamous cell carcinoma, Years of life lost, Life expectancy, Prognosis, Immunosuppression, Survival analysis

## Abstract

**Purpose:**

Cutaneous squamous cell carcinoma (cSCC) occurs predominantly in older adults. Disease-specific mortality is low, but conventional survival estimates do not capture the reduction in remaining life expectancy attributable to the disease. We therefore quantified years of life lost (YLL) in patients with cSCC overall and according to sex, age at diagnosis, progression, and risk profile.

**Methods:**

We analysed 1400 prospectively evaluated patients with primary cSCC (median age 78 years) treated at the University Hospital Tübingen. Thirty-three patients died from cSCC, 493 died from other causes, and 874 were censored. Vital status was ascertained by annual queries to the public death registry, and death-certificate information was used to support cause-of-death classification. Patients were classified as at risk if desmoplasia, bone invasion, or immunosuppression was present. Expected remaining life expectancy was derived from German cohort life tables. YLL was estimated as the difference between expected life expectancy and observed survival using empirical and Weibull distributions adjusted for age and censoring.

**Results:**

For the entire cohort, estimated YLL was 5.67 years; estimates were 5.30 years in men and 6.28 years in women. YLL was strongly influenced by age, sex, progression, and risk factors. In 70-year-old men, the estimated YLL was 7.7 years in patients with progression or risk factors and 2.5 years in those without. The corresponding estimates in women were 11.8 and 6.2 years. Although cSCC-specific mortality in the cohort was only 2.4%, the reduction in life expectancy was clinically relevant, particularly among women and high-risk patients.

**Conclusion:**

Years of life lost in cSCC increase in the presence of desmoplasia, bone invasion, immunosuppression, and tumor progression. YLL complements conventional survival measures and highlights a clinically meaningful disease burden despite low disease-specific mortality.

**Supplementary Information:**

The online version contains supplementary material available at 10.1007/s00432-026-06515-8.

## Introduction

Non-melanoma skin cancer (NMSC) is the most common malignancy in White populations, and its incidence has risen markedly over recent decades (Balkenhol et al. 2025; Olsen et al. 2024). Approximately one-third of NMSC cases are cutaneous squamous cell carcinoma (cSCC), which is usually curable but can lead to local recurrence, metastasis, and disease-specific death (Keim et al. 2023).

Prospective cohort studies from Tübingen have identified the principal prognostic determinants of adverse outcome in cSCC. Increased tumor thickness, desmoplastic growth, and immunosuppression were associated with impaired tumor-specific survival (Eigentler et al. 2017). Subsequent analyses showed that cSCC-related death results from local infiltration, locoregional spread, and distant metastasis, with desmoplasia, bone invasion, and immunosuppression representing the most relevant risk factors (Eigentler et al. 2022). Perineural invasion (PNI), although infrequent, further worsens prognosis (Haug et al. 2020).

Whereas survival probabilities and cause-specific mortality have been described, years of life lost (YLL) have not been quantified in cSCC. Because most patients are diagnosed late in life (Balkenhol et al. 2025; Keim et al. 2023; Olsen et al. 2024), YLL may provide a more clinically intuitive estimate of disease burden than mortality proportions alone. We therefore estimated YLL in patients with cSCC according to sex, age at diagnosis, tumor progression, and baseline risk profile.

## Patients and methods

### Study population

This analysis is based on 1400 prospectively evaluated patients with primary cSCC treated at the University Hospital Tübingen between 1997 and 2015, as described previously (Eigentler et al. 2017, 2022). At diagnosis, no patient had evidence of locoregional or distant metastasis. All tumors underwent complete excision and three-dimensional histologic assessment. In the case of incomplete excision, re-excision was performed until R0 status was achieved.

Among these patients, 33 died from cSCC and 493 died from causes unrelated to cSCC; 874 observations were censored. As part of the Tübingen cSCC database procedures, the public death registry was contacted once annually to ascertain vital status. Information from death certificates was used to support cause-of-death classification. Patients were classified as having died from cSCC when the clinical course and death-certificate information supported progressive cSCC as the cause of death. Patients were categorized as at risk if one or more of the following factors were present at baseline: desmoplasia, bone invasion, or immunosuppression. Each of these variables had previously emerged as an independent predictor of cSCC-specific death in multivariable analyses.

Patients were divided into two groups (Table 1):

**Table 1:** Classification of patients according to progression or baseline risk

With progression or at risk	Censoring	*n*	Group	Total
0	0	362	1	1074
0	1	712		
1	0	164	2	326
1	1	162		

For patients who died, censoring was coded as 0. For patients who survived, censoring was coded as 1

- Group 1: without progression or risk (*n* = 1,074).
- Group 2: with progression and/or risk (*n* = 326).

Expected remaining life expectancy after diagnosis was obtained from the official German cohort life tables for birth years 1871–2017 (Statistisches Bundesamt 2017).

Patients were diagnosed between 15 February 1997 and 1 April 2015. Follow-up ended on 14 December 2015, the date of the last documented cSCC-specific death in the cohort.

The study was approved by the independent ethics committee of the University Hospital of Tübingen (reference number 568/2016BO1) and was conducted in accordance with the STROBE statement for observational studies. The patients provided written informed consent on a voluntary basis.

### Statistical analysis

Years of life lost were calculated as the difference between expected remaining life expectancy in the German general population and estimated mean survival after cSCC diagnosis: YLL = LE_expected − S_mean. Expected remaining life expectancy was derived from German cohort life tables stratified by sex and age. Mean survival after diagnosis was estimated using Weibull survival models with a fixed shape parameter of beta = 2. Scale parameters were estimated by maximum likelihood within strata defined by risk group, sex, and age group. Mean survival was calculated as alpha × Gamma(1.5), where alpha is the Weibull scale parameter.

For deceased patients, the likelihood contribution was the Weibull density; for censored patients, the likelihood contribution was the Weibull survival function. The analysis therefore used all-cause survival with censoring adjustment and did not estimate YLL by directly summing years lost only among patients certified as having died from cSCC.

We also calculated the percentage of life lost (PLL) and the relative life lost (RLL), defined as YLL divided by age at death. This additional ratio was used to avoid inflation of PLL estimates in younger age groups.

Analyses were performed separately for men and women and for each age-at-diagnosis stratum. Model fit was assessed by overlaying empirical and Weibull distributions. All analyses were performed with JMP version 17.2 (SAS Institute Inc., Cary, NC, USA).

## Results

### Baseline and survival characteristics

The median age at diagnosis was 78 years (range 27–101 years), with men presenting on average 4 years earlier than women. Among the 1400 patients, 124 (8.9%) developed tumor progression. Progression was documented in all 33 patients with cSCC-specific death, in 42 of 493 patients who died from other causes (8.5%), and in 49 of 874 censored patients (5.6%). For the entire cohort, the estimated mean survival was 5.29 years, whereas the expected remaining life expectancy was 10.96 years, resulting in an estimated YLL of 5.67 years. The corresponding YLL estimates were 5.30 years for men and 6.28 years for women. Detailed age-, sex-, and group-specific numbers of patients, censored observations, deaths from other causes, cSCC-specific deaths, and YLL estimates are provided in Supplementary Tables 1, 2 and 3.

Among censored patients, 168 had their final observation within the last year of follow-up and 706 had their final observation more than one year before study termination; the corresponding proportions are shown in Supplementary Table 4.

### Years of life lost in patients without progression or baseline risk factors

**Fig. 1:**
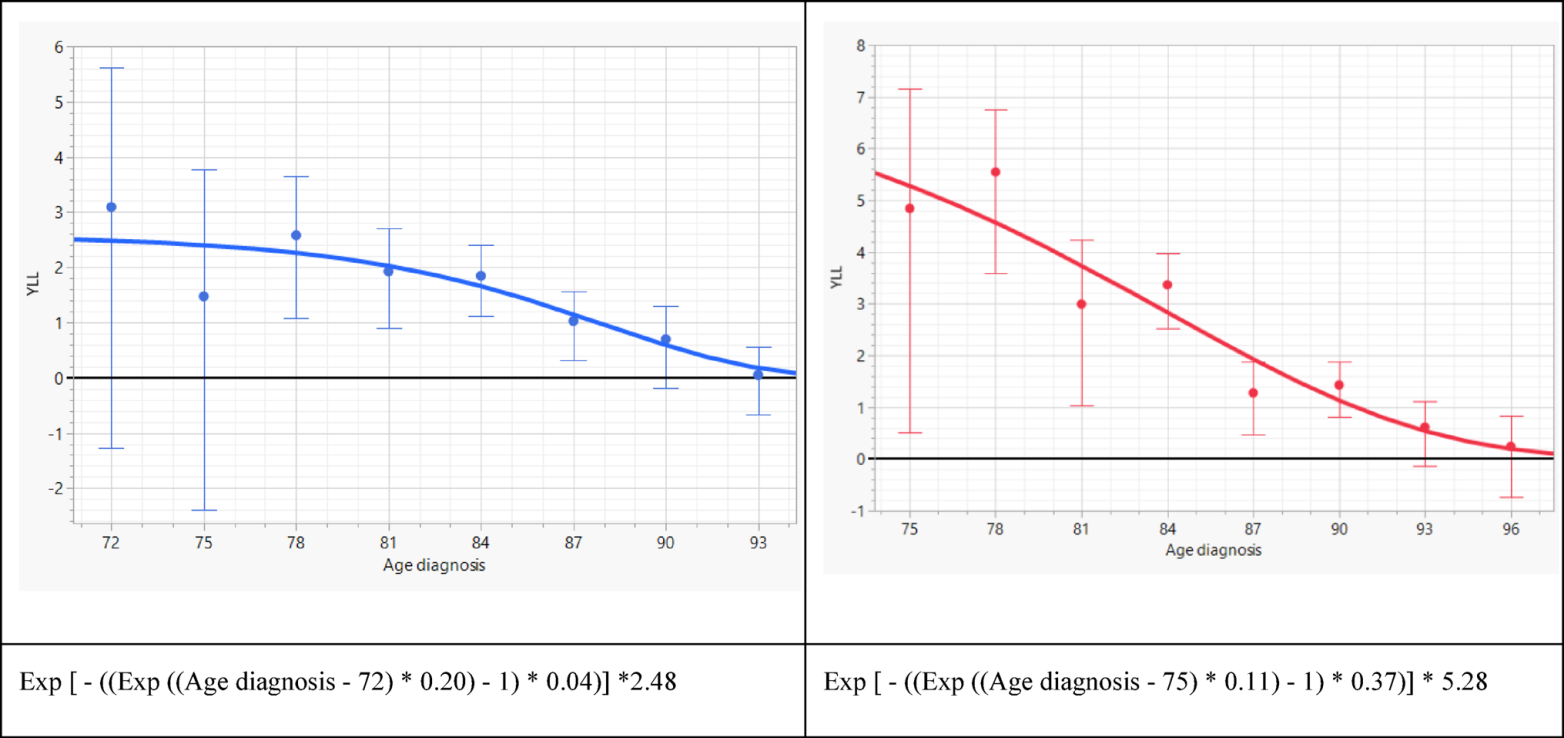
Years of life lost in males without progression or baseline risk (left) and in females without progression or baseline risk (right)

Among patients without progression or predefined risk factors, estimated YLL declined substantially with increasing age at diagnosis. In men, YLL was 2.4 years at age 75, 1.5 years at age 85, and 0.6 years at age 90 (Fig. 1). In women, the corresponding losses were higher, reaching 5.3 years at age 75, 4.0 years at age 80, and 1.1 years at age 90. The fitted Weibull models showed close agreement with the observed survival distributions (Supplementary Fig. 1A, B).

### Years of life lost in patients with progression or risk factors

**Fig. 2:**
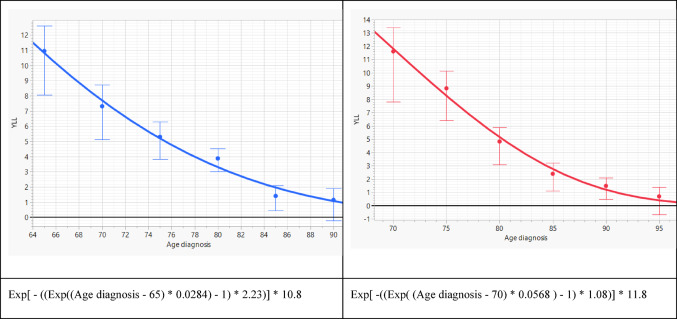
Years of life lost in males with progression or baseline risk (left) and in females with progression or baseline risk (right)

In contrast, patients with tumor progression or at least one prognostic risk factor—desmoplasia, bone invasion, or immunosuppression—showed substantially higher YLL. In men, the estimated losses were 10.8 years at age 65, 5.2 years at age 75, and 2.0 years at age 85 (Fig. 2). Women again showed higher YLL, with estimates of 11.8 years at age 70, 5.2 years at age 80, and 1.2 years at age 90. Across all analyses, the Weibull models fitted the empirical survival data closely (Supplementary Fig. 2A, B).

### Comparative analyses

**Fig. 3:**
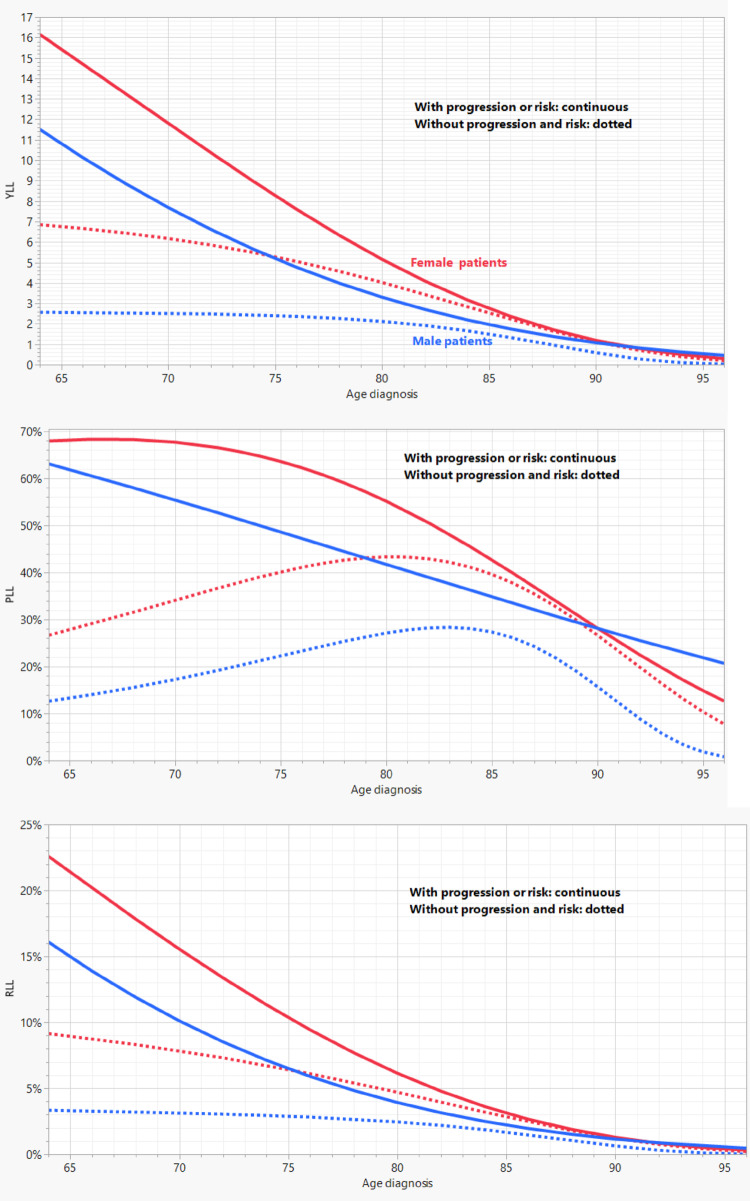
Overlay plots for years of life lost (upper), percentage of life lost (middle), and relative life lost (lower)

Aggregated analyses (Fig. 3) demonstrated a consistent sex effect, with women showing greater YLL across age categories. The same pattern was observed for PLL and RLL.

Compared with stage II-III cutaneous melanoma reported by Vikström et al. (2024), YLL values in cSCC were of similar magnitude in older patients (Table 2), despite the generally lower biological aggressiveness of cSCC.

**Table 2:**
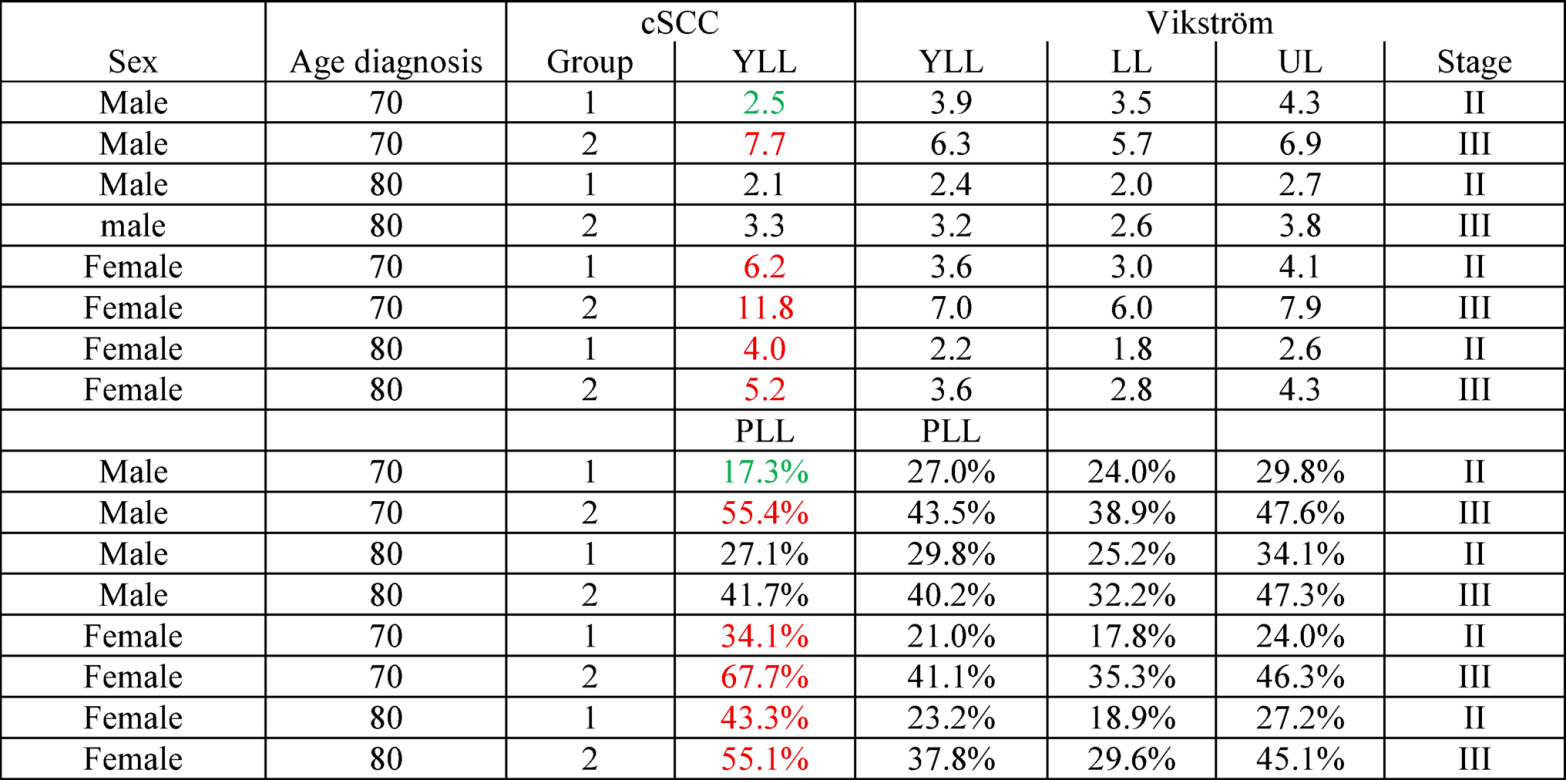
Comparison of cSCC data with the melanoma data reported by Vikström et al. (2024)

Values are shown in black if they fall within the confidence interval reported by Vikström et al. (2024), in green if they are below the interval, and in red if they are above the interval

## Discussion

To our knowledge, this is the first study to quantify years of life lost in patients with cutaneous squamous cell carcinoma. Although disease-specific mortality was low in this cohort (2.4%), cSCC was associated with a measurable reduction in remaining life expectancy, particularly in patients with progression, desmoplasia, bone invasion, or immunosuppression.

The YLL estimates should not be interpreted as a direct summation of years lost among the 33 patients who died from cSCC. Rather, they are model-based estimates of reduced remaining life expectancy in defined patient strata, using all-cause survival and censoring-adjusted estimation.

These findings extend the prognostic framework established in prior Tübingen studies. Eigentler et al. (2017) showed that desmoplasia and immunosuppression adversely affect tumor-specific survival, and Eigentler et al. (2022) demonstrated that desmoplasia, bone invasion, and immunosuppression are the leading determinants of cSCC-related death. Haug et al. (2020) further showed that PNI aggravates outcome, mainly in combination with other high-risk features. The present analysis translates these prognostic patterns into a clinically intuitive measure of lost life expectancy.

Women in this cohort had longer expected survival than men, but also a greater absolute and relative life loss once affected by cSCC. This observation probably reflects the higher remaining life expectancy of women in the reference population rather than more aggressive tumor biology. The RLL metric may therefore be helpful when counselling older patients because it expresses the burden of cSCC relative to the lifespan otherwise expected at that age.

The YLL patterns are also consistent with the previously described modes of cSCC-related death, namely local infiltration, locoregional spread, and distant metastasis (Eigentler et al. 2022). The good fit of the Weibull models across subgroups supports the internal consistency of the estimated survival trajectories.

The comparison with melanoma is noteworthy. In older patients, YLL in cSCC reached a magnitude similar to that reported for stage II-III melanoma (Vikström et al. 2024), despite the much lower case-fatality rate of cSCC. This underlines that YLL adds clinically relevant context beyond conventional mortality measures.

### Limitations

This study has several limitations. First, YLL estimation required extrapolation beyond the observed follow-up period. Second, the cohort originated from a single German tertiary centre, which may limit generalisability. Third, treatment advances after 2015, including the broader use of systemic immunotherapies for advanced cSCC, were not captured. Finally, only the three strongest clinicopathologic risk factors were used for stratification; other relevant variables such as tumor thickness and PNI were not incorporated into the final grouping model. Because this was a long-term observational cohort of elderly patients, censoring and potential uncertainty in cause-of-death attribution remain relevant limitations. However, vital status was checked annually through the public death registry, death-certificate information was used for cause-of-death classification, and all patients classified as having died from cSCC had documented tumor progression. Some age-, sex-, and risk-specific strata contained few cSCC-specific deaths; the resulting YLL estimates should therefore be interpreted as model-based estimates with greater uncertainty in smaller subgroups.

### Clinical implications

YLL and RLL may improve communication of prognosis in elderly patients with cSCC by translating risk factors into expected loss of lifespan. Patients with desmoplastic tumors, bone invasion, or immunosuppression appear to warrant particularly close surveillance and may be appropriate candidates for intensified multidisciplinary management.

## Conclusions

In this prospective cSCC cohort, the average years of life lost were modest in absolute terms but increased markedly in the presence of histologic risk factors or tumor progression. Women showed higher relative life loss despite later diagnosis. YLL complements standard survival metrics and helps to quantify the hidden burden of cSCC in an elderly population.

## Supplementary Information

Below is the link to the electronic supplementary material.

Supplementary Material 1

Supplementary Material 2

## Data Availability

The datasets generated and/or analysed during the current study are available from the corresponding author on reasonable request.
