# Supplementary material for: Years of life lost in cutaneous squamous cell carcinoma: analysis of a prospective cohort of 1400 patients
Source: J Cancer Res Clin Oncol. 2026 May 25;152(5):113. doi: 10.1007/s00432-026-06515-8 (PMC13212848; doi:10.1007/s00432-026-06515-8)
Supplement: Supplementary file 1 — Supplementary Material 1 [file 432_2026_6515_MOESM1_ESM.docx]

**Supplementary Material**

Years of life lost in cutaneous squamous cell carcinoma: analysis of a prospective cohort of 1,400 patients

**Supplementary methods**

Vital status was ascertained as part of the prospectively maintained Tübingen cSCC database. The public death registry was contacted once annually, and information from death certificates was used to support cause-of-death classification.

Years of life lost (YLL) were calculated as YLL = LE_expected - S_mean, where LE_expected is the expected remaining life expectancy from German cohort life tables stratified by sex and age, and S_mean is the estimated mean survival after cSCC diagnosis. Survival after diagnosis was estimated using Weibull distributions with a fixed shape parameter beta = 2. For deceased patients, the likelihood contribution was the Weibull density; for censored patients, the likelihood contribution was the Weibull survival function. Scale parameters were estimated by maximum likelihood within strata defined by risk group, sex, and age group. Mean survival was calculated as alpha x Gamma(1.5).

Group 1 comprised patients without progression and without desmoplasia, bone invasion, or immunosuppression. Group 2 comprised patients with progression and/or at least one of these risk factors.

**Supplementary Table 1. Stratum-specific YLL and event counts in Group 1.**

| **Group** | **sex** | **Age groups** | **Mean survival (years)** | **Mean (years to live)** | **YLL** | **n** | **n censored** | **% censored** | **n died** | **Death by other cause** | **death by cSCC** |
| --- | --- | --- | --- | --- | --- | --- | --- | --- | --- | --- | --- |
| 1 | male | 72 | 9.86 | 13.0 | 3.09 | 77 | 68 | 88.3% | 9 | 9 | 0 |
| 1 | male | 75 | 9.38 | 10.9 | 1.47 | 96 | 86 | 89.6% | 10 | 10 | 0 |
| 1 | male | 78 | 6.30 | 8.9 | 2.58 | 83 | 59 | 71.1% | 24 | 24 | 0 |
| 1 | male | 81 | 5.40 | 7.3 | 1.93 | 100 | 65 | 65.0% | 35 | 35 | 0 |
| 1 | male | 84 | 4.06 | 5.9 | 1.85 | 80 | 40 | 50.0% | 40 | 40 | 0 |
| 1 | male | 87 | 3.65 | 4.7 | 1.03 | 54 | 20 | 37.0% | 34 | 34 | 0 |
| 1 | male | 90 | 3.13 | 3.8 | 0.70 | 26 | 8 | 30.8% | 18 | 18 | 0 |
| 1 | male | 93 | 2.89 | 2.9 | 0.05 | 25 | 3 | 12.0% | 22 | 22 | 0 |
| 1 | female | 75 | 8.23 | 13.1 | 4.84 | 33 | 26 | 78.8% | 7 | 7 | 0 |
| 1 | female | 78 | 5.32 | 10.9 | 5.54 | 40 | 28 | 70.0% | 12 | 12 | 0 |
| 1 | female | 81 | 5.65 | 8.6 | 2.99 | 43 | 30 | 69.8% | 13 | 13 | 0 |
| 1 | female | 84 | 3.53 | 6.9 | 3.37 | 40 | 16 | 40.0% | 24 | 24 | 0 |
| 1 | female | 87 | 4.06 | 5.3 | 1.28 | 51 | 18 | 35.3% | 33 | 33 | 0 |
| 1 | female | 90 | 2.82 | 4.2 | 1.43 | 33 | 5 | 15.2% | 28 | 28 | 0 |
| 1 | female | 93 | 2.53 | 3.1 | 0.61 | 21 | 4 | 19.0% | 17 | 17 | 0 |
| 1 | female | 96 | 2.38 | 2.6 | 0.24 | 11 | 1 | 9.1% | 10 | 10 | 0 |

**Supplementary Table 2. Stratum-specific YLL and event counts in Group 2.**

| **Group** | **sex** | **Age groups** | **Mean survival (years)** | **Mean (years to live)** | **YLL** | **n** | **n censored** | **% censored** | **n died** | **Death by other cause** | **death by cSCC** |
| --- | --- | --- | --- | --- | --- | --- | --- | --- | --- | --- | --- |
| 2 | male | 65 | 6.50 | 17.4 | 10.95 | 24 | 15 | 62.5% | 9 | 8 | 1 |
| 2 | male | 70 | 6.61 | 13.9 | 7.32 | 38 | 24 | 63.2% | 14 | 10 | 4 |
| 2 | male | 75 | 5.47 | 10.8 | 5.30 | 48 | 28 | 58.3% | 20 | 14 | 6 |
| 2 | male | 80 | 3.98 | 7.9 | 3.88 | 47 | 19 | 40.4% | 28 | 23 | 5 |
| 2 | male | 85 | 4.06 | 5.5 | 1.41 | 39 | 14 | 35.9% | 25 | 19 | 6 |
| 2 | male | 90 | 2.83 | 4.0 | 1.15 | 13 | 5 | 38.5% | 8 | 7 | 1 |
| 2 | female | 70 | 5.65 | 17.3 | 11.61 | 13 | 8 | 61.5% | 5 | 4 | 1 |
| 2 | female | 75 | 4.62 | 13.5 | 8.83 | 14 | 7 | 50.0% | 7 | 5 | 2 |
| 2 | female | 80 | 4.48 | 9.3 | 4.83 | 19 | 8 | 42.1% | 11 | 10 | 1 |
| 2 | female | 85 | 3.70 | 6.1 | 2.40 | 18 | 5 | 27.8% | 13 | 12 | 1 |
| 2 | female | 90 | 2.58 | 4.1 | 1.48 | 12 | 1 | 8.3% | 11 | 9 | 2 |
| 2 | female | 95 | 2.34 | 3.0 | 0.69 | 6 | 0 | 0.0% | 6 | 5 | 1 |

**Supplementary Table 3. Overall YLL and event counts for the entire cohort.**

| **sex** | **Age groups** | **Mean survival (years)** | **Mean (years to live)** | **YLL** | **n** | **n censored** | **% censored** | **n died** | **Death by other cause** | **death by cSCC** |
| --- | --- | --- | --- | --- | --- | --- | --- | --- | --- | --- |
| males | 27 - 99 | 5.74 | 11.04 | 5.30 | 938 | 621 | 66.2% | 317 | 292 | 25 |
| females | 34 - 101 | 4.53 | 10.81 | 6.28 | 462 | 253 | 54.8% | 209 | 201 | 8 |
| all | 27 - 101 | 5.29 | 10.96 | 5.67 | 1400 | 874 | 62.4% | 526 | 493 | 33 |

**Supplementary Table 4. Final state in the last year of follow-up and prior to the last year.**

| **Final state** | **Last year of study** | **Prior to last year** | **Total** | **% prior to last year** |
| --- | --- | --- | --- | --- |
| Alive or censored | 168 | 706 | 874 | 80.8% |
| Death by cSCC | 13 | 20 | 33 | 60.6% |
| Death by other cause | 34 | 459 | 493 | 93.1% |

Abbreviations: cSCC, cutaneous squamous cell carcinoma; YLL, years of life lost; LE, life expectancy.
