# Supplementary material for: Years of life lost in cutaneous squamous cell carcinoma: analysis of a prospective cohort of 1400 patients
Source: J Cancer Res Clin Oncol. 2026 May 25;152(5):113. doi: 10.1007/s00432-026-06515-8 (PMC13212848; doi:10.1007/s00432-026-06515-8)

Supplementary Fig. 1A Weibull distribution for each age group in males without progression or baseline risk


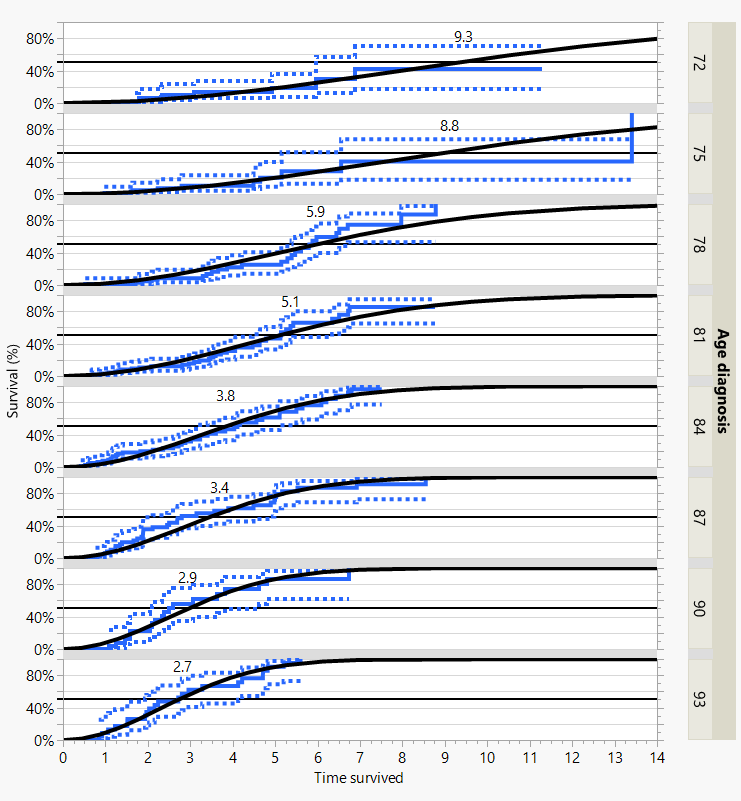


Supplementary Fig. 1B Weibull distribution for each age group in females without progression or baseline risk


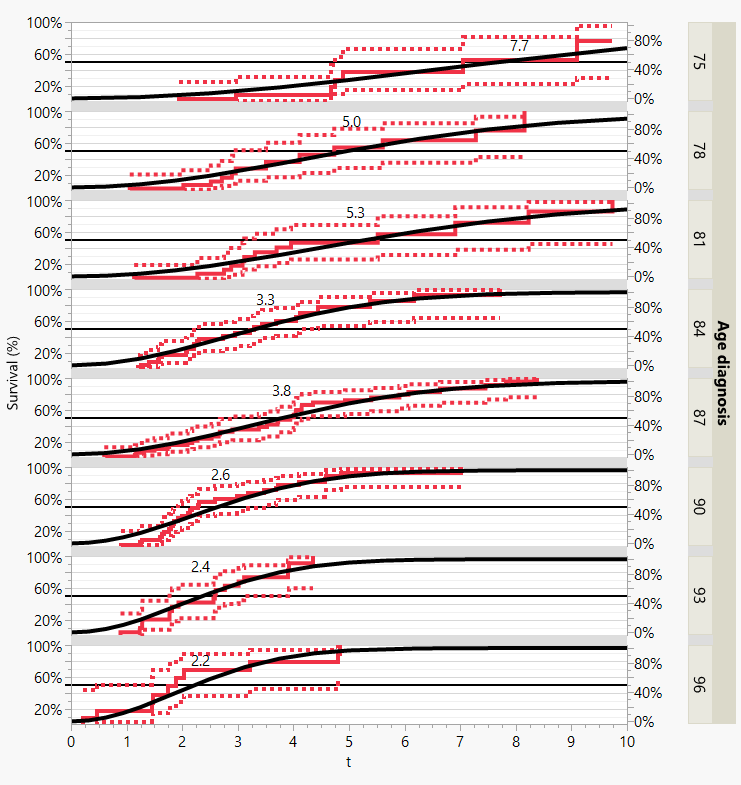


Supplementary Fig. 2A Weibull distribution for each age group in males with progression or baseline risk


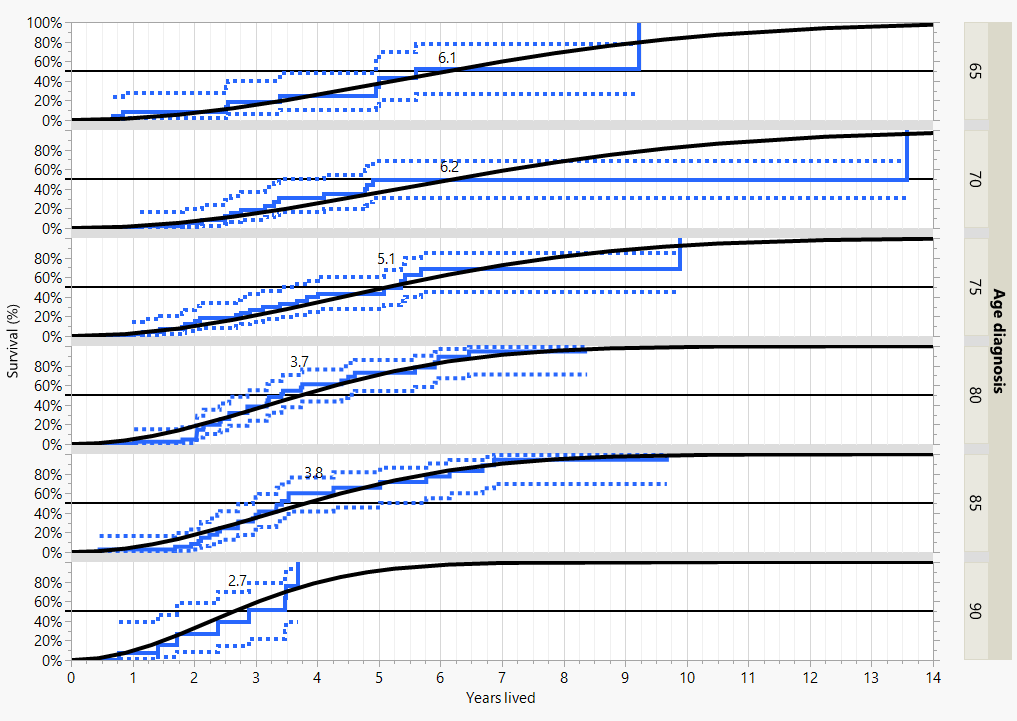


Supplementary Fig. 2B Weibull distribution for each age group in females with progression or baseline risk


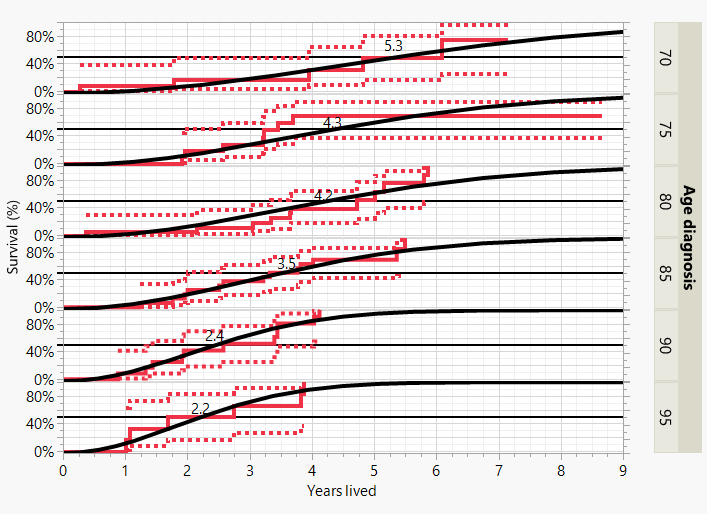

Supplement: Supplementary file 2 — Supplementary Material 2 [file 432_2026_6515_MOESM2_ESM.docx]
